# Pathological and MRI-Based Infiltrative Markers Show Distinct Associations with Oncologic Outcomes in Undifferentiated Pleomorphic Sarcoma and Myxofibrosarcoma

**DOI:** 10.3390/curroncol33100572

**Published:** 2026-09-22

**Authors:** Akane Ariga, Yuki Funauchi, Kyoko Yamashita, Masanori Saito, Keiko Hayakawa, Taisuke Tanizawa, Norio Kurosawa, Toshitaka Yoshii, Keisuke Ae

**Affiliations:** 1 Department of Orthopaedic Surgery, Institute of Science Tokyo, Tokyo 113-8510, Japan; ariga.akane@tmd.ac.jp (A.A.); norio.kurosawa@jfcr.or.jp (N.K.); yoshii.orth@tmd.ac.jp (T.Y.); 2 Department of Orthopaedic Surgery, Cancer Institute Hospital of JFCR, Tokyo 135-8550, Japan; masanori.saito@jfcr.or.jp (M.S.); keiko.hayakawa@jfcr.or.jp (K.H.); taisuke.tanizawa@jfcr.or.jp (T.T.); keisuke.ae@jfcr.or.jp (K.A.); 3 Department of Pathology, Cancer Institute Hospital of JFCR, Tokyo 135-8550, Japan; kyoko.yamashita@jfcr.or.jp

**Keywords:** undifferentiated pleomorphic sarcoma, myxofibrosarcoma, infiltrative growth, magnetic resonance imaging, tail sign, pathological infiltration, progression-free survival

## Abstract

Undifferentiated pleomorphic sarcoma (UPS) and myxofibrosarcoma (MFS) frequently exhibit infiltrative growth, but whether pathological and magnetic resonance imaging (MRI)-based findings represent the same tumor characteristics remain unclear. We examined the relationships among report-documented pathological infiltration (PI), MRI-defined diffuse infiltration (DI), and radiological infiltration (RI) based on the tail sign, and explored their associations with oncologic outcomes. This single-center retrospective cohort study included 68 treatment-naïve patients with primary UPS or MFS without distant or lymph-node metastasis at presentation who underwent curative-intent resection. The primary outcome was progression-free survival (PFS), with overall survival (OS), distant metastasis-free survival (DMFS), and local recurrence-free survival (LRFS) evaluated as secondary outcomes. Time-to-event associations were evaluated using Firth’s penalized Cox regression with parsimonious covariate adjustment and sensitivity analyses restricted to patients with R0 resection. DI, RI, and PI were observed in 75.0%, 76.5%, and 60.3% of patients, respectively. RI positivity was more frequent among patients with PI, whereas DI showed no clear relationship with PI or RI. In an exploratory multivariable model, DI (hazard ratio [HR], 3.30; 95% confidence interval [CI], 1.05–16.51; *p* = 0.040) and RI (HR, 4.26; 95% CI, 1.31–22.29; *p* = 0.013) were associated with worse PFS. Local recurrences occurred only in patients with PI, although adjusted estimates were imprecise. These findings suggest that pathological and MRI-based infiltrative markers may capture different and potentially complementary aspects of tumor behavior. External validation using standardized imaging and pathological review is required.

## Simple Summary

Undifferentiated pleomorphic sarcoma and myxofibrosarcoma are rare soft-tissue malignancies that can spread microscopically into nearby tissues, making their true extent difficult to assess. We compared three common indicators of infiltrative growth: one based on pathology specimens and two based on preoperative magnetic resonance imaging. Among 68 patients who underwent surgery, the imaging indicators did not fully match the pathological indicator. The two imaging indicators were associated with a higher risk of disease progression, whereas local recurrence occurred only in patients with pathological infiltration. These findings suggest that pathology and magnetic resonance imaging describe different aspects of tumor spread rather than the same phenomenon. Larger multicenter studies using standardized imaging and pathology review are needed before these indicators can be used to guide treatment or follow-up.

## 1. Introduction

Soft-tissue sarcomas (STSs) are a heterogeneous group of rare malignant tumors of mesenchymal origin that exhibit substantial variation in histological subtype, tumor grade, patterns of local invasion, and metastatic potential [1,2,3]. Advances in preoperative local assessment using magnetic resonance imaging (MRI), multidisciplinary management at specialized sarcoma centers, appropriate surgical resection, and radiotherapy have improved treatment outcomes [2,4]. Nevertheless, local recurrence and distant metastasis remain major clinical challenges, particularly in patients with high-grade STSs [2,5]. Accurate stratification of the risks of local treatment failure and systemic disease progression is therefore essential for optimizing treatment strategies.

Undifferentiated pleomorphic sarcoma (UPS) and myxofibrosarcoma (MFS) are adult STS subtypes that frequently exhibit infiltrative growth into the surrounding tissues [5,6,7,8,9,10,11]. These tumors may show microscopic extensions beyond the main tumor mass, including extension along fascial planes, as well as the tail sign on MRI, which is characterized by a linear or tapering extension contiguous with the primary tumor [8,9,10,11]. Consequently, assessment based solely on the gross tumor mass may underestimate the true extent of disease [6,7,8,9,10,11]. Such irregular extension into the surrounding tissues may complicate delineation of the tumor extent, affect surgical and radiotherapy planning, and contribute to the risk of local treatment failure [6,7,8,9,10,11,12].

In previous studies, infiltrative growth in UPS and MFS has been characterized using pathological [13] and MRI-based approaches [6,7,8,14]. Pathological infiltration (PI) refers to irregular microscopic extension of tumor cells beyond the main tumor border into adjacent adipose tissue, fascia, skeletal muscle, or other surrounding structures on histopathological examination [6,7,11]. An MRI-based pattern of diffuse infiltration has been defined as an indistinct tumor–host interface involving a substantial proportion of the tumor circumference, including a threshold of at least 25% in previous studies; this finding is hereafter termed diffuse infiltration (DI) [6,15]. Another MRI feature, the tail sign, is characterized by a linear or tapering extension contiguous with the main tumor and extending along a fascial plane [7,9,10,14]. In the present study, this tail-sign–based finding is termed radiological infiltration (RI).

MRI-based infiltrative findings have often been interpreted as imaging surrogates of microscopic pathological infiltration [6,7,8,9,10,11]. However, it remains unclear whether PI, DI, and RI reflect the same underlying biological process assessed using different modalities or capture distinct aspects of tumor extension and tumor–host interaction [7,11]. PI directly demonstrates microscopic tumor-cell extension at the tumor–host interface, whereas DI may also reflect peritumoral edema, inflammation, stromal remodeling, or altered vascular permeability [6,10,11]. RI, in contrast, depicts a relatively localized linear or tapering extension, predominantly along fascial planes [10,16]. These differences raise the possibility that the three markers may provide distinct and potentially complementary information regarding the risks of local recurrence and systemic disease progression.

In the present study, we evaluated PI, DI, and RI within the same cohort of patients with primary UPS or MFS who had no distant or lymph node metastases at presentation and underwent initial curative-intent wide resection. We investigated the relationships among these infiltrative markers, their associations with clinicopathological characteristics, and their associations with overall survival (OS), local recurrence-free survival (LRFS), distant metastasis-free survival (DMFS), and progression-free survival (PFS). The aim of this study was to determine whether pathological and MRI-based infiltrative markers reflect the same underlying biological process or show distinct relationships with clinicopathological characteristics and oncologic outcomes.

## 2. Materials and Methods

### 2.1. Study Design and Patient Selection

This retrospective observational study was conducted at a single tertiary cancer center. We screened consecutive patients who underwent initial curative-intent wide resection for primary soft-tissue sarcoma at The Cancer Institute Hospital of the Japanese Foundation for Cancer Research between October 2017 and November 2023. Eligible patients had no evidence of distant or lymph node metastasis at initial presentation. To focus on histological subtypes characterized by infiltrative growth [1,2,6,7], only patients with a final pathological diagnosis of undifferentiated pleomorphic sarcoma (UPS) or myxofibrosarcoma (MFS) were included.

Patients were excluded if they had a history of surgery, preoperative chemotherapy, or preoperative radiotherapy for the index tumor; had undergone unplanned excision at another institution; or underwent surgery for recurrent disease. Additional exclusion criteria were the inability to evaluate preoperative contrast-enhanced magnetic resonance imaging (MRI), inability to assess pathological findings in the resected specimen, or inability to determine the presence or absence of infiltrative growth from the final pathological report. Ultimately, 68 treatment-naïve patients with primary UPS or MFS who underwent initial curative-intent wide resection at our institution were included in the analysis (Figure 1).

The duration of follow-up was not included in the eligibility criteria. All patients who met the inclusion criteria were included regardless of follow-up length. Patients without an event for a given endpoint were censored on the date of the last confirmed event-free follow-up. Accordingly, patients with less than 2 years of postoperative follow-up were included in the survival analyses and were censored at their last follow-up visit.

Postoperative radiotherapy (RT) was not an exclusion criterion, as various subsequent therapies could be administered depending on tumor behavior, margin status, and subsequent oncologic outcomes, and we considered these events to provide essential information. Information regarding postoperative RT—including the irradiated site, indication, dose and fractionation, and timing relative to surgery—was retrospectively extracted from clinical records. RT delivered to the primary tumor site as postoperative adjuvant local therapy was distinguished from RT subsequently delivered to metastatic lesions following disease progression.

Among 543 patients who underwent first curative-intent wide resection for primary soft-tissue sarcoma, 124 had a final pathological diagnosis of undifferentiated pleomorphic sarcoma (UPS) or myxofibrosarcoma (MFS). After exclusion of patients with distant or lymph node metastasis at presentation and those meeting the other prespecified exclusion criteria, 68 patients (UPS, n = 40; MFS, n = 28) were included in the final analysis. MRI, magnetic resonance imaging.

### 2.2. Assessment of Pathological Infiltration

Pathological infiltration (PI) was assessed according to the concept of infiltrative growth reported in previous studies [6,7,11,13,16]. PI was retrospectively determined from the final pathology reports prepared by board-certified pathologists for the resected specimens. Cases were classified as PI-positive when the final pathological report explicitly described irregular extension of tumor cells beyond the tumor margin into the surrounding adipose tissue, fascia, skeletal muscle, or other adjacent structures [7,11]. Cases without an explicit description of infiltrative growth in the final pathology report were classified as PI-negative.

The original histological slides were not re-reviewed specifically for PI classification in this study, and no centralized or standardized histopathological reassessment was performed. Accordingly, PI in this study represents report-documented pathological infiltration rather than infiltration determined by a standardized central histopathological review. PI classification was performed without reference to MRI-defined diffuse infiltration (DI), radiological infiltration (RI) based on the tail sign, or postoperative oncologic outcomes.

### 2.3. Assessment of MRI-Based Infiltration

Preoperative magnetic resonance imaging (MRI) was used to evaluate two MRI-based infiltrative markers: diffuse infiltration (DI) and radiological infiltration (RI). For all 68 patients, DI and RI were assessed on fat-suppressed contrast-enhanced T1-weighted images (CE FS T1WI). The median interval between the MRI examination used for assessment and surgery was 15 days (range, 3–69 days). Because the MRI examinations were acquired as part of routine clinical care between 2017 and 2023, a single prospectively standardized acquisition protocol was not used throughout the study period. Thus, scanner platforms and detailed acquisition parameters may have varied among patients, although CE FS T1WI was available and used for assessment in all cases. Only preoperative MRI examinations were used. Owing to the retrospective nature of the study, the temporal relationship between the MRI examination used for assessment and biopsy could not be reliably established for all patients.

When axial, coronal, and sagittal images were all available, all three imaging planes were assessed. If images in all three planes were unavailable, at least two orthogonal planes were evaluated. For each available imaging plane, the single image showing the largest cross-sectional area of the tumor was selected. DI and RI were assessed on each of these selected images, and a marker was classified as positive if its predefined criteria were met in at least one imaging plane.

DI was defined with reference to the criteria described by Fernebro et al. and Nakamura et al. as a diffuse infiltrative tumor margin characterized by an indistinct interface between the tumor and surrounding tissues involving at least 25% of the tumor circumference on any of the selected images (Figure 2A) [6,15].

RI was defined with reference to the criteria reported by Kaya et al. and Iwata et al. as the presence of the tail sign, characterized by a linear or tapering contrast-enhancing extension contiguous with the primary tumor and extending along a fascial plane on any of the selected images (Figure 2B) [7,14].

DI and RI were evaluated independently. DI was assessed as a diffuse infiltrative change involving the tumor margin, whereas RI was assessed as a linear or tapering extension predominantly along a fascial plane. All MRI examinations were reviewed by a single orthopedic oncologist with more than 10 years of experience in musculoskeletal oncology, who was blinded to the PI classification and postoperative oncologic outcomes. A second independent reader was not available for the present cohort, and formal interobserver agreement was therefore not assessed.

### 2.4. Assessment of Surgical Margin

Surgical margin status was determined based on the final pathological report and classified as microscopically negative (R0) or microscopically positive (R1). R0 resection was defined as the absence of tumor cells at the resection margin on histopathological examination, whereas R1 resection was defined as the presence of microscopic tumor cells at the resection margin. No patients in the study cohort underwent R2 resection, defined as macroscopic residual tumor after surgery.

The association between surgical margin status and each infiltrative marker was evaluated. Surgical margin status was also included as a covariate in the time-to-event analyses. In addition, sensitivity analyses restricted to patients who underwent R0 resection were performed to assess the potential influence of surgical margin status on the study outcomes.

### 2.5. Outcomes

The primary outcome was progression-free survival (PFS). PFS was defined as the time from the date of wide resection to the first occurrence of local recurrence, distant metastasis, or death from any cause. Patients who remained free of these events were censored on the date of their last confirmed event-free follow-up.

Secondary outcomes included overall survival (OS), distant metastasis-free survival (DMFS), and local recurrence-free survival (LRFS). OS was defined as the time from the date of wide resection to death from any cause, with surviving patients censored at the date of the last confirmed follow-up. DMFS was defined as the time from the date of wide resection to the first diagnosis of distant metastasis, with patients without distant metastasis censored at the date of the last confirmed metastasis-free follow-up. LRFS was defined as the time from the date of wide resection to the first diagnosis of local recurrence, with patients without local recurrence censored at the date of the last confirmed local recurrence-free follow-up.

PFS was selected as the primary outcome to provide an integrated measure of overall disease progression. LRFS and DMFS were evaluated as secondary component-specific outcomes to distinguish associations with local treatment failure from those with systemic disease progression, which are combined within the composite PFS endpoint. Given the limited numbers of local recurrence and distant metastasis events, analyses of LRFS and DMFS were considered exploratory.

### 2.6. Statistical Analysis

Continuous variables were summarized as the mean and standard deviation (SD) or the median and interquartile range (IQR), as appropriate according to their distribution. Categorical variables were summarized as frequencies and percentages. There were no missing data for the variables included in the analyses.

The associations among DI, RI, and PI, the relationships between each infiltrative marker and pathological surgical margin status, and comparisons of crude event frequencies according to infiltrative marker status were evaluated using Fisher’s exact test. Exploratory associations between each binary infiltrative marker and clinicopathological characteristics were evaluated using univariable logistic regression, with results reported as odds ratios (ORs) and 95% confidence intervals (CIs). Continuous variables were entered on their original scale. Nominal categorical variables were modeled using prespecified reference categories, whereas FNCLCC histological grade was treated as an ordered categorical variable and modeled using orthogonal polynomial contrasts. Tumor size was evaluated both as a continuous variable and using prespecified cutoff values of 5 and 10 cm. Because these analyses were exploratory and involved multiple clinicopathological variables, interpretation emphasized effect estimates and 95% CIs.

Time-to-event distributions were estimated using the Kaplan–Meier method and compared using the log-rank test. Median follow-up was estimated using the reverse Kaplan–Meier method [17]. Associations with overall survival (OS), local recurrence-free survival (LRFS), distant metastasis-free survival (DMFS), and progression-free survival (PFS) were quantified using hazard ratios (HRs) and 95% CIs. Given the limited number of events and the possibility of complete or quasi-complete separation [18], Firth’s penalized Cox proportional hazards regression was used throughout the survival analyses.

PFS was the primary endpoint, whereas OS, DMFS, and LRFS were secondary endpoints; analyses of DMFS and LRFS were considered exploratory because of the limited numbers of events. Each infiltrative marker was first evaluated separately in univariable Firth Cox models for each endpoint. To compare DI, RI, and PI within a common analytical framework and to avoid selective omission of individual markers, complementary exploratory three-marker models including DI, RI, and PI simultaneously were then fitted for each endpoint. Additional exploratory common models incorporating pathological surgical margin status were used to assess whether the observed associations could be explained by microscopically positive surgical margins. These common models were intended to provide a shared framework for comparison of the three infiltrative markers rather than to establish their independent prognostic value. Results from the univariable and common multivariable models are presented in Tables S1–S3.

Given the limited number of events, outcome-specific adjusted models were also constructed using parsimonious sets of clinically relevant covariates and were interpreted as exploratory adjusted analyses. The PFS model included DI and RI with adjustment for age, FNCLCC histological grade, tumor size, and pathological surgical margin status. The OS and DMFS models evaluated DI with adjustment for the same covariates, whereas the exploratory LRFS model evaluated PI with adjustment for tumor size and pathological surgical margin status. Sensitivity analyses restricted to patients who underwent R0 resection were performed using the corresponding reduced covariate sets.

All statistical tests were two-sided, and *p* < 0.05 was considered statistically significant. Because multiple exploratory analyses were performed, no formal adjustment for multiple comparisons was applied. Interpretation therefore emphasized effect estimates, 95% CIs, and the consistency of findings across analyses rather than individual *p* values alone. Estimates derived from analyses with few events or wide 95% CIs were interpreted cautiously as hypothesis-generating findings. All statistical analyses were performed using R version 4.3.2 (R Foundation for Statistical Computing, Vienna, Austria).

## 3. Results

### 3.1. Patient Characteristics

A total of 68 patients were included in the final analysis (Figure 1). Histological subtypes comprised 40 patients (58.8%) with undifferentiated pleomorphic sarcoma (UPS) and 28 (41.2%) with myxofibrosarcoma (MFS). The mean age was 68.5 years (SD, 12.3 years), and 39 patients (57.4%) were male. Forty-seven patients (69.1%) had FNCLCC grade 3 tumors, and the mean tumor size was 7.5 cm (SD, 3.8 cm). Deep-seated tumors were present in 46 patients (67.6%), and 50 tumors (73.5%) arose in the extremities. Pathological surgical margin status was classified as R0 in 65 patients (95.6%) and R1 in 3 patients (4.4%) (Table 1).

The median follow-up, estimated using the reverse Kaplan–Meier method, was 56.4 months (95% CI, 48.9–63.3 months). During follow-up, 20 patients died, 22 developed distant metastasis, and 7 experienced local recurrence. Of the 20 deaths, 19 were sarcoma-related and one was attributed to old age. A total of 27 patients experienced a PFS event, defined as the first occurrence of local recurrence, distant metastasis, or death from any cause.

Seven of the 68 patients received radiotherapy (RT) at some point after surgery. Of these, two patients (2.9%) received postoperative adjuvant RT to the primary tumor site, whereas the remaining five received RT for metastatic lesions after disease progression. Both patients who received primary-site postoperative RT had MFS arising in a deep extremity location and were positive for DI, RI, and PI. Both were classified as R0 according to the final pathological margin variable used in the analysis; however, clinical records documented concern regarding possible focal margin involvement. Infiltrative growth was also considered in the decision to administer RT in one of the two patients. Both patients received 66 Gy in 33 fractions, initiated approximately 3.5 months after surgery, and neither developed local recurrence during follow-up. Given that only two patients received primary-site postoperative RT, no formal subgroup comparison or RT-adjusted survival analysis was performed.

### 3.2. Associations Among Infiltration Markers

DI, RI, and PI were present in 51 (75.0%), 52 (76.5%), and 41 patients (60.3%), respectively. Patients with RI had higher odds of being PI-positive than those without RI (OR, 3.43; 95% CI, 0.93–13.33; *p* = 0.043). In contrast, DI was not associated with either RI (OR, 1.51; 95% CI, 0.34–5.96; *p* = 0.523) or PI (OR, 1.08; 95% CI, 0.30–3.78; *p* = 1.000). None of the infiltrative markers showed a clear association with pathological surgical margin status (Table 2).

### 3.3. Exploratory Clinicopathological Correlates of Infiltration Markers

Exploratory univariable associations between the infiltrative markers and clinicopathological characteristics are summarized in Table 3. PI positivity differed according to histological subtype and was more frequent in MFS than in UPS. With MFS as the reference category, UPS was associated with lower odds of PI positivity (OR, 0.25; 95% CI, 0.08–0.74; *p* = 0.012). Increasing tumor size was also associated with lower odds of PI positivity (OR per 1 cm increase, 0.85; 95% CI, 0.74–0.98; *p* = 0.029). When tumor size was dichotomized at 5 cm, tumors larger than 5 cm had lower odds of PI positivity than tumors measuring ≤ 5 cm (OR, 0.15; 95% CI, 0.03–0.75; *p* = 0.020). No clear univariable associations were identified between DI or RI and the clinicopathological characteristics presented in Table 3.

### 3.4. Crude Event Frequencies According to Infiltration Markers

In the descriptive analysis of crude event frequencies, which did not account for differences in follow-up duration, patients with DI had higher crude frequencies of death, distant metastasis, and PFS events than those without DI (Table 4). Death occurred in 19 of 51 patients (37.3%) with DI and in 1 of 17 patients (5.9%) without DI (*p* = 0.015). Distant metastasis occurred in 20 of 51 patients (39.2%) with DI and in 2 of 17 patients (11.8%) without DI (*p* = 0.041). PFS events occurred in 25 of 51 patients (49.0%) with DI and in 2 of 17 patients (11.8%) without DI (*p* = 0.009).

Patients with RI also had a higher crude frequency of PFS events than those without RI (25/52 [48.1%] vs. 2/16 [12.5%]; *p* = 0.018). Although death and distant metastasis were also more frequent among patients with RI, the between-group differences did not reach statistical significance. No clear differences in the crude frequencies of death or distant metastasis were observed according to PI status. In contrast, local recurrence occurred in 7 of 41 patients (17.1%) with PI, whereas no local recurrences were observed among the 27 patients without PI (*p* = 0.037).

### 3.5. Survival Analyses

#### 3.5.1. Progression-Free Survival

Kaplan–Meier analysis showed that patients with DI had shorter PFS than those without DI (log-rank *p* = 0.010; Figure 3A). Similarly, patients with RI had shorter PFS than those without RI (log-rank *p* = 0.021; Figure 3B).

In univariable Firth Cox analyses, DI (HR, 4.37; 95% CI, 1.43–21.57; *p* = 0.007) and RI (HR, 3.80; 95% CI, 1.24–18.74; *p* = 0.016) were associated with worse PFS, whereas PI showed no clear association (HR, 1.28; 95% CI, 0.60–2.92; *p* = 0.534) (Table S1). In the common model including DI, RI, and PI simultaneously, DI (HR, 4.12; 95% CI, 1.35–20.33; *p* = 0.010) and RI (HR, 3.53; 95% CI, 1.12–17.68; *p* = 0.029) remained associated with worse PFS, whereas PI did not (HR, 1.00; 95% CI, 0.46–2.31; *p* = 0.998) (Table S2). After pathological surgical margin status was added to this model, DI (HR, 3.83; 95% CI, 1.24–18.96; *p* = 0.017) and RI (HR, 4.86; 95% CI, 1.41–26.01; *p* = 0.009) remained associated with worse PFS, whereas PI showed no clear association (HR, 0.88; 95% CI, 0.40–2.07; *p* = 0.767) (Table S3).

In the outcome-specific model adjusted for age, FNCLCC histological grade, tumor size, and pathological surgical margin status, DI (HR, 3.30; 95% CI, 1.05–16.51; *p* = 0.040) and RI (HR, 4.26; 95% CI, 1.31–22.29; *p* = 0.013) remained associated with worse PFS (Table 5). In the sensitivity analysis restricted to patients who underwent R0 resection, DI remained associated with worse PFS (HR, 3.44; 95% CI, 1.09–17.18; *p* = 0.033). RI showed an association in the direction of worse PFS, although the confidence interval included the null value (HR, 2.94; 95% CI, 0.95–14.61; *p* = 0.064) (Table S4).

#### 3.5.2. Overall Survival and Distant Metastasis-Free Survival

Kaplan–Meier analysis showed that patients with DI had shorter OS (log-rank *p* = 0.019; Figure S1) and DMFS (log-rank *p* = 0.036; Figure S2) than those without DI. In univariable Firth Cox analyses, DI was associated with worse OS (HR, 5.22; 95% CI, 1.33–47.09; *p* = 0.014) and worse DMFS (HR, 3.43; 95% CI, 1.10–17.07; *p* = 0.032) (Table S1).

In the common three-marker model, DI remained associated with worse OS (HR, 4.95; 95% CI, 1.26–44.78; *p* = 0.018) and DMFS (HR, 3.21; 95% CI, 1.02–16.00; *p* = 0.045), whereas RI and PI showed no clear associations with either endpoint (Table S2). After pathological surgical margin status was added, DI remained associated with worse OS (HR, 4.87; 95% CI, 1.23–44.20; *p* = 0.021). For DMFS, the estimate for DI was attenuated and the confidence interval included the null value (HR, 2.92; 95% CI, 0.92–14.62; *p* = 0.072), whereas RI was associated with worse DMFS (HR, 3.97; 95% CI, 1.16–20.89; *p* = 0.026) (Table S3). These common models were exploratory and should not be interpreted as definitive comparisons among markers.

In the outcome-specific models adjusted for age, FNCLCC histological grade, tumor size, and pathological surgical margin status, the association between DI and OS did not reach statistical significance (HR, 3.78; 95% CI, 0.95–34.28; *p* = 0.061). DI also showed an association in the direction of worse DMFS, although the confidence interval included the null value (HR, 2.69; 95% CI, 0.83–13.62; *p* = 0.104) (Table 5).

#### 3.5.3. Local Recurrence-Free Survival

Kaplan–Meier analysis showed that patients with PI had shorter LRFS than those without PI (log-rank *p* = 0.033; Figure 3C). Because only seven local recurrence events were observed, all subsequent LRFS analyses were considered exploratory.

In univariable Firth Cox analyses, PI was associated with worse LRFS (HR, 9.78; 95% CI, 1.19–1269.13; *p* = 0.030). RI had a large effect estimate, but the confidence interval was extremely wide and included the null value (HR, 5.12; 95% CI, 0.62–665.61; *p* = 0.154); DI showed no clear association (HR, 1.75; 95% CI, 0.37–16.84; *p* = 0.513) (Table S1). In the common three-marker model, PI retained the largest effect estimate, but precision was limited (HR, 7.54; 95% CI, 0.90–986.29; *p* = 0.067) (Table S2). After pathological surgical margin status was added, the estimate remained large but highly imprecise (HR, 6.59; 95% CI, 0.77–862.51; *p* = 0.096) (Table S3).

In the outcome-specific exploratory model adjusted for tumor size and pathological surgical margin status, PI was associated with worse LRFS (HR, 9.55; 95% CI, 1.06–1270.53; *p* = 0.043) (Table 5). A similar finding was observed in the Kaplan–Meier analysis restricted to patients who underwent R0 resection (log-rank *p* = 0.041; Figure S3). In the R0-restricted sensitivity model adjusted for tumor size, PI remained associated with worse LRFS (HR, 10.31; 95% CI, 1.12–1385.04; *p* = 0.037) (Table S4). However, only seven local recurrences occurred, all in PI-positive tumors, and the corresponding effect estimates were highly imprecise, as reflected by the extremely wide confidence intervals. Accordingly, the association between PI and LRFS should be regarded as exploratory and hypothesis-generating rather than as evidence that PI is an independently validated predictor of local recurrence.

## 4. Discussion

In this retrospective study of patients with UPS and MFS who presented without distant or lymph node metastases and underwent initial curative-intent wide resection, we directly compared pathological infiltration (PI) with two MRI-based infiltrative markers, diffuse infiltration (DI) and radiological infiltration (RI), within the same cohort. Three principal findings emerged. First, DI and RI were associated with worse PFS in the common three-marker models and in the outcome-specific model adjusted for clinicopathological factors, whereas PI showed no clear association with PFS. Second, all seven local recurrences occurred in tumors positive for both PI and RI; PI showed the clearest association with LRFS, although the small number of events and complete overlap between PI and RI among recurrent cases precluded reliable comparison of their respective roles. Third, RI was associated with PI, whereas DI showed no clear association with either PI or RI. These findings suggest that PI, DI, and RI may capture distinct, potentially complementary aspects of infiltrative tumor behavior rather than representing a single process assessed by different methods.

DI was associated with higher crude frequencies of death, distant metastasis, and PFS events, and with worse OS, DMFS, and PFS in univariable analyses. In the common three-marker models, DI remained associated with these outcomes, although adjustment for pathological surgical margin status attenuated the DMFS estimate. In the outcome-specific clinicopathologically adjusted models, DI remained associated with worse PFS, whereas its associations with OS and DMFS did not reach statistical significance. These findings do not establish DI as an independently validated prognostic factor, but they support the possibility that DI provides information regarding the overall risk of disease progression, consistent with previous reports linking infiltrative MRI growth patterns to adverse oncologic outcomes in soft-tissue sarcoma [6,15].

The absence of a clear association between DI and PI suggests that a diffusely infiltrative MRI margin may not directly correspond to microscopic tumor-cell infiltration documented histopathologically [6,8,10,11]. DI may instead reflect a broader tumor–host interface incorporating tumor extension together with peritumoral edema, inflammation, stromal alteration, altered vascular permeability, necrosis, or hemorrhage. By contrast, RI represents a relatively localized linear or tapering extension, predominantly along fascial planes, and its association with PI is consistent with previous descriptions of the tail sign [7,8,9,10,11,14]. RI was also associated with PFS in the common and outcome-specific models. Thus, DI and RI may reflect different spatial patterns of MRI-defined infiltration, while PI directly reflects report-documented microscopic extension. Because imaging findings were not spatially correlated with corresponding histopathological or molecular features, these biological interpretations remain hypothesis-generating.

PI showed no clear association with death or distant metastasis, whereas local recurrence occurred exclusively in PI-positive tumors. The association remained evident after adjustment for pathological surgical margin status and in the R0-restricted analysis, suggesting that it was not explained solely by microscopically positive margins. These findings raise the possibility that report-documented PI captures aspects of local infiltrative behavior not fully represented by conventional pathological margin status. However, only seven local recurrences occurred, all in tumors positive for both PI and RI. Although Firth’s penalized Cox regression reduces small-sample bias and permits estimation in sparse or separated data, it cannot overcome the limited information provided by so few events [18]. The confidence intervals were therefore very wide, and PI should not be regarded as an independently validated predictor of local recurrence. The PI–LRFS association is exploratory and hypothesis-generating and requires external validation.

PI positivity was more frequent in MFS than in UPS, consistent with the recognized morphology of MFS, including irregular extension along fascial planes and into surrounding tissues [1,3,7,8,9,10,14]. Histological subtype may therefore have contributed to the observed association between PI and local recurrence. With only seven local recurrence events, PI and histological subtype could not be reliably separated in a fully adjusted LRFS model or adequately powered subtype-specific analyses; residual confounding by histological subtype cannot be excluded. An inverse univariable association between tumor size and PI positivity was also observed. This unexpected finding may reflect differences in histological subtype, tumor morphology, pathological sampling, or residual confounding and should not be interpreted as an independent association.

The statistical models were designed for complementary purposes. The outcome-specific adjusted PFS model was the main exploratory adjusted analysis for the primary endpoint, using a limited number of clinically relevant covariates to reduce overfitting. The common models allowed DI, RI, and PI to be compared within the same analytical framework, whereas analyses of OS, DMFS, and LRFS were secondary and exploratory. The very small number of local recurrence events precluded a comparably complex LRFS model with reliable precision. Accordingly, all multivariable analyses should be interpreted as exploratory adjusted analyses rather than confirmatory assessments of independent prognostic value, particularly because multiple exploratory comparisons were performed without formal multiplicity adjustment.

Taken together, the findings highlight the limitations of treating infiltrative features as a single entity. PI evaluates microscopic tumor extension documented on histopathological examination, RI primarily assesses linear or tapering extension along a fascial plane, and DI evaluates broader MRI alterations at the tumor margin [6,7,8,9,10,11,14,15]. If externally validated, combined assessment of these features may provide a more comprehensive characterization of local invasiveness and overall disease progression than any single marker alone.

From a surgical perspective, infiltrative soft-tissue sarcoma may complicate the balance between oncologically appropriate resection and functional preservation. Contemporary reviews and recent MFS series emphasize the challenges posed by infiltrative growth, margin achievement, and local control [19,20,21,22,23,24,25,26,27,28]. Multidisciplinary preoperative planning, including reconstructive expertise when appropriate, may facilitate margin-oriented resection and reconstruction when substantial soft-tissue defects are anticipated [19]. The contribution of perioperative radiotherapy to local control in MFS also remains difficult to separate from margin status and treatment selection [23,24,25,26,27,28]. However, the present study did not evaluate marker-guided surgical or radiotherapy strategies, and our findings do not support modifying surgical margins, reconstructive strategies, radiotherapy, postoperative surveillance, or other adjuvant treatment solely on the basis of PI, DI, or RI.

This study has several limitations. First, it was a retrospective single-center study of 68 patients, with limited numbers of outcome events, particularly only seven local recurrences. This restricted multivariable modeling and resulted in very wide confidence intervals despite Firth’s penalized Cox regression [18]. The outcome-specific models differed in covariate composition, the common three-marker models were complementary exploratory analyses, and no formal adjustment for multiple comparisons was performed. Second, PI was determined retrospectively from the original final pathology reports rather than by standardized centralized histopathological review [29,30]. Reporting thresholds and terminology may have varied among pathologists and over time; therefore, PI-negative status indicates absence of explicitly documented infiltration rather than definitive absence of microscopic infiltration.

Third, DI and RI were assessed on fat-suppressed contrast-enhanced T1-weighted images by a single experienced reader, without formal evaluation of interobserver reproducibility [9,10,31]. MRI examinations were acquired during routine clinical care over several years, and scanner platforms and detailed acquisition parameters were not prospectively standardized. The temporal relationship between biopsy and the MRI examination could not be reliably established for all patients, so biopsy-related changes may have influenced MRI appearance; postoperative changes could not have affected classification because only preoperative MRI was used. Fourth, only two patients received postoperative adjuvant RT to the primary tumor site. Both were DI-, RI-, and PI-positive and were treated in the context of clinical concern regarding possible focal margin involvement, introducing potential confounding by indication. The small number of RT-treated patients and local recurrence events precluded an informative RT-adjusted or RT-excluded LRFS analysis.

Finally, only three patients underwent R1 resection, limiting assessment of the independent effect of pathological margin status; both margin-adjusted and R0-restricted analyses should therefore be considered exploratory. UPS and MFS are biologically distinct histological subtypes despite shared infiltrative characteristics [1,2,3], and the limited number of local recurrences precluded adequately adjusted subtype-specific analyses. In addition, although 19 of 20 deaths were sarcoma-related, PFS and OS used death from any cause, and no cause-specific survival analysis was prespecified.

Despite these limitations, this study directly compared PI, DI, and RI within the same cohort and evaluated their interrelationships and associations with distinct oncologic outcomes. RI was associated with PI, whereas DI showed no clear association with PI. DI and RI were associated with PFS, while PI showed its clearest relationship with local recurrence. These findings suggest that pathological and MRI-based infiltrative markers may capture distinct aspects of oncologic behavior and provide complementary information. Future studies should validate these observations in larger multicenter cohorts using standardized central imaging and pathological review, formal interobserver assessment, adequately powered subtype-specific analyses, and spatial correlation of imaging findings with histopathological and molecular characteristics.

## 5. Conclusions

In this retrospective single-center study of patients with primary undifferentiated pleomorphic sarcoma or myxofibrosarcoma without distant or lymph-node metastasis at presentation, pathological and MRI-based infiltrative markers were not fully concordant and showed distinct associations with oncologic outcomes. DI and RI were associated with worse PFS, suggesting potential relevance to the overall risk of disease progression, whereas PI showed its clearest association with local recurrence. Local recurrence occurred exclusively in patients with report-documented PI, and the association between PI and LRFS remained evident in the R0-restricted analysis; however, this finding was based on only seven local recurrence events, with highly imprecise effect estimates, and should therefore be regarded as exploratory and hypothesis-generating rather than evidence of independently validated prognostic value. Taken together, these findings suggest that DI, RI, and PI may capture complementary aspects of tumor behavior, with potentially different relationships to local treatment failure and overall disease progression. The present findings do not support modification of surgical margins, radiotherapy, surveillance, or adjuvant treatment solely on the basis of PI, DI, or RI. Larger multicenter studies incorporating standardized MRI assessment, central pathological review, interobserver evaluation, and direct radiologic–pathologic spatial correlation are warranted to validate these findings and clarify their biological and clinical significance.

## Figures and Tables

**Figure 1:**
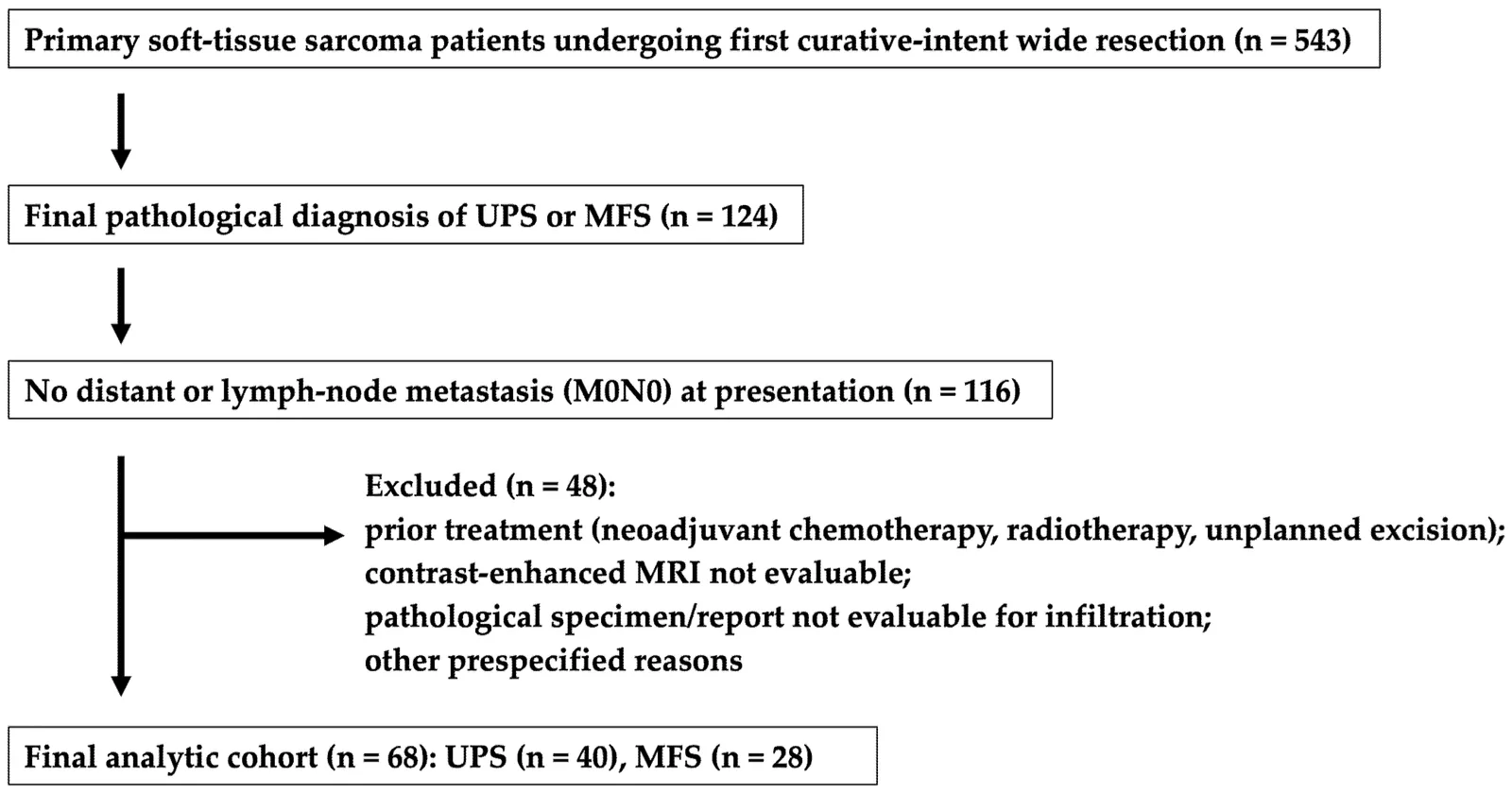
Flowchart of patient selection.

**Figure 2:**
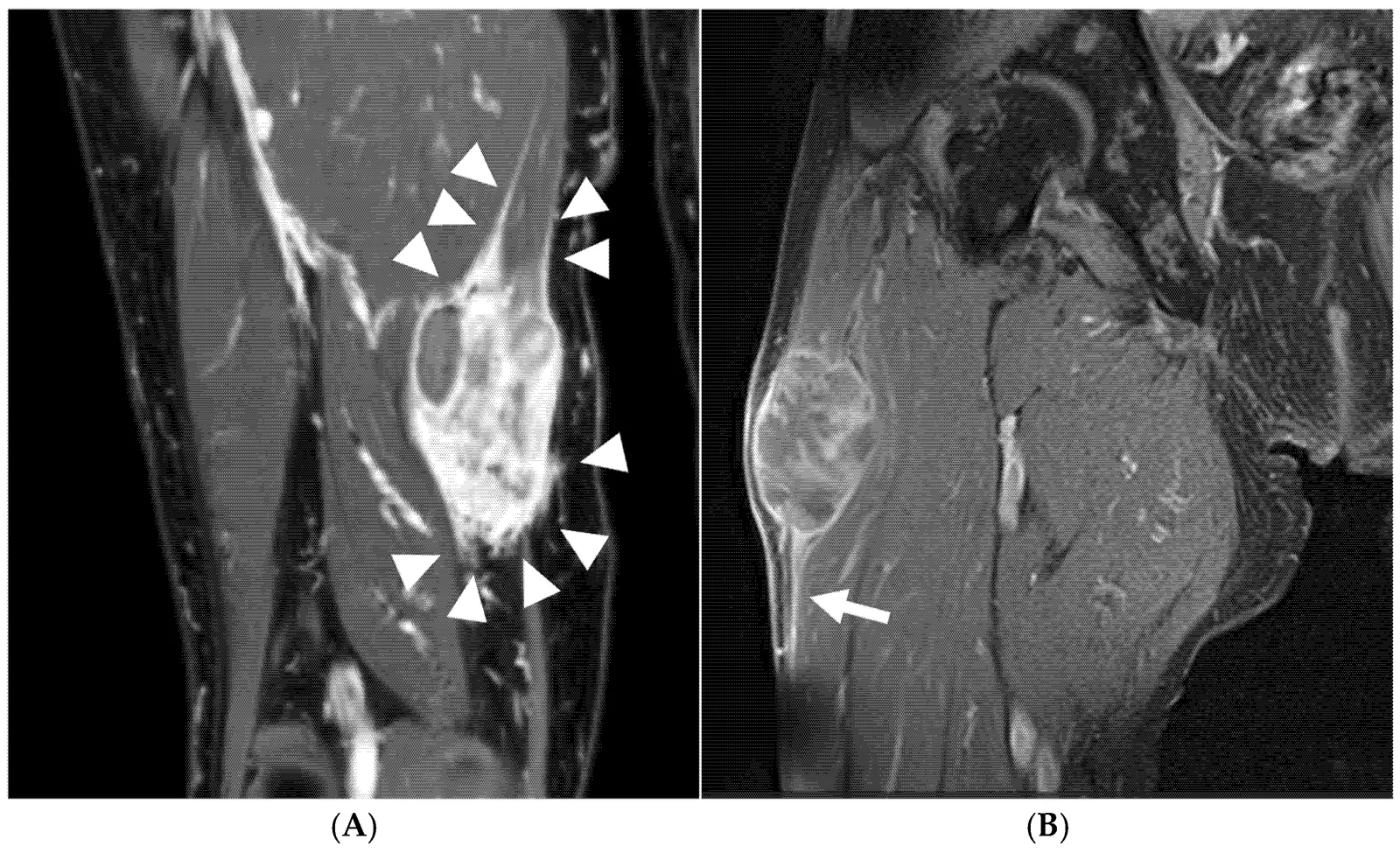
Representative fat-suppressed contrast-enhanced T1-weighted magnetic resonance images showing diffuse infiltration and radiological infiltration. (**A**) Coronal fat-suppressed contrast-enhanced T1-weighted image of an undifferentiated pleomorphic sarcoma (UPS) arising in the right thigh. Arrowheads indicate a broad, indistinct tumor–host interface involving at least 25% of the tumor circumference, classified as diffuse infiltration (DI). (**B**) Coronal fat-suppressed contrast-enhanced T1-weighted image of an UPS arising in the right thigh. The arrow indicates a linear or tapering enhancing extension contiguous with the main tumor and extending along a fascial plane, corresponding to the tail sign and classified as radiological infiltration (RI).

**Figure 3:**
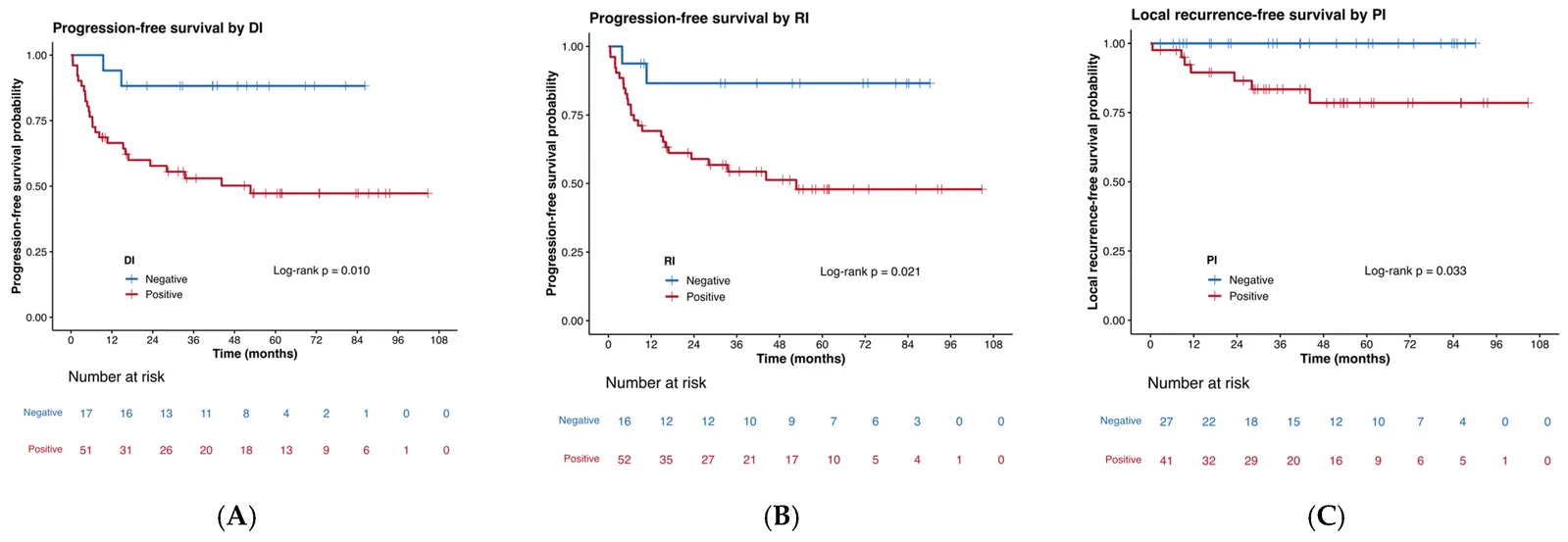
Kaplan–Meier estimates of progression-free survival and local recurrence-free survival according to infiltration status. (**A**) Progression-free survival according to diffuse infiltration (DI) status. Patients with DI-positive tumors had shorter progression-free survival than those with DI-negative tumors (log-rank *p* = 0.010). (**B**) Progression-free survival according to radiological infiltration (RI) status. Patients with RI-positive tumors had shorter progression-free survival than those with RI-negative tumors (log-rank *p* = 0.021). (**C**) Local recurrence-free survival according to pathological infiltration (PI) status. Patients with PI-positive tumors had shorter local recurrence-free survival than those with PI-negative tumors (log-rank *p* = 0.033). Tick marks indicate censored observations. Numbers at risk are shown below each panel.

**Table 1:** Baseline characteristics of the study cohort.

Characteristic	Overall Cohort (n = 68)
Age, years, mean (SD)	68.5 (12.3)
Range	33–91
Sex	
Female	29 (42.6)
Male	39 (57.4)
Histological subtype	
Myxofibrosarcoma	28 (41.2)
Undifferentiated pleomorphic sarcoma	40 (58.8)
FNCLCC grade	
1	3 (4.4)
2	18 (26.5)
3	47 (69.1)
Tumor size, cm, mean (SD)	7.5 (3.8)
Range	0.7–18.1
Tumor size category	
≤5 cm	16 (23.5)
>5 to ≤10 cm	40 (58.8)
>10 cm	12 (17.6)
Depth	
Deep	46 (67.6)
Superficial	22 (32.4)
Site	
Limb	50 (73.5)
Trunk	18 (26.5)
Surgical margin	
R0	65 (95.6)
R1	3 (4.4)
Diffuse infiltration (DI) positive	51 (75.0)
Radiological infiltration (RI) positive	52 (76.5)
Pathological infiltration (PI) positive	41 (60.3)

Footnote. Values are n (%) unless otherwise specified. Age and tumor size are presented as mean (standard deviation [SD]) and range. Tumor-size categories are mutually exclusive. FNCLCC, Fédération Nationale des Centres de Lutte Contre le Cancer; R0, microscopically negative margin; R1, microscopically positive margin.

**Table 2:** Associations among infiltration markers and surgical margin.

Comparison	Cross-Tabulation	OR (95% CI)	*p* Value
DI vs. RI	DI−: RI− 5, RI+ 12; DI+: RI− 11, RI+ 40	1.51 (0.34–5.96)	0.523
DI vs. PI	DI−: PI− 7, PI+ 10; DI+: PI− 20, PI+ 31	1.08 (0.30–3.78)	1.000
RI vs. PI	RI−: PI− 10, PI+ 6; RI+: PI− 17, PI+ 35	3.43 (0.93–13.33)	0.043
DI vs. margin	DI−: R0 17, R1 0; DI+: R0 48, R1 3	Not estimable	0.568
RI vs. margin	RI−: R0 15, R1 1; RI+: R0 50, R1 2	0.60 (0.030–37.73)	0.559
PI vs. margin	PI−: R0 27, R1 0; PI+: R0 38, R1 3	Not estimable	0.271

Footnote. Odds ratios (ORs) represent the odds of the second variable being positive (or R1) in patients positive versus negative for the first infiltration marker. ORs and exact conditional 95% confidence intervals (CIs) were estimated using Fisher’s exact test. Two-sided *p* values were calculated using Fisher’s exact test. ORs were not estimable when a zero cell precluded a finite estimate. DI, diffuse infiltration; RI, radiological infiltration; PI, pathological infiltration; R0, microscopically negative margin; R1, microscopically positive margin.

**Table 3:** Exploratory univariable associations between clinicopathological characteristics and infiltration markers.

Characteristic	Comparison	DI, OR (95% CI)	*p* Value	RI, OR (95% CI)	*p* Value	PI, OR (95% CI)	*p* Value
Age	Per 1-year increase	1.02 (0.98–1.07)	0.336	1.01 (0.97–1.06)	0.662	0.98 (0.94–1.02)	0.275
Sex	Male vs. Female	0.66 (0.21–2.07)	0.480	0.76 (0.24–2.39)	0.635	0.68 (0.25–1.84)	0.449
Histology	UPS vs. MFS	2.62 (0.85–8.06)	0.093	0.57 (0.17–1.88)	0.359	0.25 (0.08–0.74)	0.012
Tumor size	Per 1 cm increase	1.01 (0.87–1.17)	0.919	0.96 (0.83–1.11)	0.554	0.85 (0.74–0.98)	0.029
Tumor size	>5 cm vs. ≤5 cm	0.63 (0.15–2.53)	0.512	1.11 (0.30–4.09)	0.874	0.15 (0.03–0.75)	0.020
Tumor size	>10 cm vs. ≤10 cm	1.00 (0.24–4.22)	1.000	0.55 (0.14–2.12)	0.382	0.40 (0.11–1.41)	0.154
Tumor depth	Superficial vs. Deep	0.84 (0.26–2.67)	0.765	1.07 (0.32–3.57)	0.914	1.65 (0.57–4.81)	0.360
Tumor site	Trunk vs. Extremity	1.23 (0.34–4.42)	0.751	1.11 (0.31–4.00)	0.879	0.77 (0.26–2.28)	0.632

Footnote. FNCLCC histological grade is not presented because it was modeled as an ordered categorical variable using orthogonal polynomial contrasts, yielding separate linear and quadratic contrast estimates that are not directly interpretable as comparisons between individual grade categories. Pathological surgical margin status is presented separately in Table 2. All analyses were exploratory and univariable. OR, odds ratio; CI, confidence interval; DI, diffuse infiltration; RI, radiological infiltration; PI, pathological infiltration; UPS, undifferentiated pleomorphic sarcoma; MFS, myxofibrosarcoma; FNCLCC, Fédération Nationale des Centres de Lutte Contre le Cancer.

**Table 4:** Event frequencies according to infiltration markers.

Outcome	Marker	Negative, n/N (%)	Positive, n/N (%)	*p* Value
Death	DI	1/17 (5.9)	19/51 (37.3)	0.015
Death	RI	2/16 (12.5)	18/52 (34.6)	0.121
Death	PI	7/27 (25.9)	13/41 (31.7)	0.786
Local recurrence	DI	1/17 (5.9)	6/51 (11.8)	0.670
Local recurrence	RI	0/16 (0.0)	7/52 (13.5)	0.186
Local recurrence	PI	0/27 (0.0)	7/41 (17.1)	0.037
Distant metastasis	DI	2/17 (11.8)	20/51 (39.2)	0.041
Distant metastasis	RI	2/16 (12.5)	20/52 (38.5)	0.069
Distant metastasis	PI	9/27 (33.3)	13/41 (31.7)	1.000
PFS event	DI	2/17 (11.8)	25/51 (49.0)	0.009
PFS event	RI	2/16 (12.5)	25/52 (48.1)	0.018
PFS event	PI	9/27 (33.3)	18/41 (43.9)	0.453

Footnote. Values are events/number at risk (%). *p* values were calculated using Fisher exact test. PFS, progression-free survival. These crude event proportions do not account for differences in follow-up duration.

**Table 5:** Outcome-specific parsimonious Firth penalized Cox regression models.

Endpoint	Model	Variable	HR	95% CI	*p* Value
OS	Age + grade + size + margin + DI	DI	3.78	0.95–34.28	0.061
DMFS	Age + grade + size + margin + DI	DI	2.69	0.83–13.62	0.104
PFS	Age + grade + size + margin + DI + RI	DI	3.30	1.05–16.51	0.040
PFS	Age + grade + size + margin + DI + RI	RI	4.26	1.31–22.29	0.013
LRFS	Size + margin (exploratory)	PI	9.55	1.06–1270.53	0.043

Footnote. Only the focal infiltrative-marker coefficients are shown; all variables listed in the Model column were included in the corresponding model. The LRFS model was exploratory because only seven local recurrence events occurred. CI, confidence interval; DI, diffuse infiltration; DMFS, distant metastasis-free survival; HR, hazard ratio; LRFS, local recurrence-free survival; OS, overall survival; PFS, progression-free survival; RI, radiological infiltration; PI, pathological infiltration.

## Data Availability

The data presented in this study are available from the corresponding author upon reasonable request. The data are not publicly available because they contain information that could compromise patient privacy and are subject to institutional ethical restrictions.

## Supplementary Materials

The following supporting information can be downloaded at: https://www.mdpi.com/article/10.3390/curroncol33100572/s1, Table S1: Univariable Firth penalized Cox regression analyses of infiltration markers; Table S2: Exploratory common three-marker Firth penalized Cox regression models; Table S3: Exploratory common three-marker models additionally adjusted for pathological surgical margin status; Table S4: Sensitivity analyses restricted to patients with R0 resection; Figure S1: Overall survival according to diffuse infiltration; Figure S2: Distant metastasis-free survival according to diffuse infiltration; Figure S3: Local recurrence-free survival according to pathological infiltration in patients with R0 resection.

## Abbreviations

The following abbreviations are used in this manuscript:

CI	Confidence interval
DI	Diffuse infiltration
DMFS	Distant metastasis-free survival
FNCLCC	Fédération Nationale des Centres de Lutte Contre le Cancer
HR	Hazard ratio
IQR	Interquartile range
LRFS	Local recurrence-free survival
MFS	Myxofibrosarcoma
MRI	Magnetic resonance imaging
OR	Odds ratio
OS	Overall survival
PFS	Progression-free survival
PI	Pathological infiltration
RI	Radiological infiltration
SD	Standard deviation
STS	Soft tissue sarcoma
UPS	Undifferentiated pleomorphic sarcoma
