# Supplementary material for: Pathological and MRI-Based Infiltrative Markers Show Distinct Associations with Oncologic Outcomes in Undifferentiated Pleomorphic Sarcoma and Myxofibrosarcoma
Source: Curr Oncol. 2026 Sep 22;33(10):572. doi: 10.3390/curroncol33100572 (PMC13703746; doi:10.3390/curroncol33100572)

Table S1. Univariable Firth penalized Cox regression analyses of infiltration markers.

Endpoint	Marker	HR	95% CI	p Value
OS	DI	5.22	1.33–47.09	0.014
OS	RI	2.59	0.82–12.96	0.115
OS	PI	1.18	0.49–3.05	0.713
LRFS	DI	1.75	0.37–16.84	0.513
LRFS	RI	5.12	0.62–665.61	0.154
LRFS	PI	9.78	1.19–1269.13	0.030
DMFS	DI	3.43	1.10–17.07	0.032
DMFS	RI	2.89	0.93–14.40	0.070
DMFS	PI	0.92	0.40–2.17	0.836
PFS	DI	4.37	1.43–21.57	0.007
PFS	RI	3.80	1.24–18.74	0.016
PFS	PI	1.28	0.60–2.92	0.534

Footnote. Each marker was evaluated in a separate univariable model. Estimates for LRFS should be interpreted with particular caution because only seven local recurrence events occurred. CI, confidence interval; DI, diffuse infiltration; DMFS, distant metastasis-free survival; HR, hazard ratio; LRFS, local recurrence-free survival; OS, overall survival; PFS, progression-free survival; RI, radiological infiltration; PI, pathological infiltration.

Table S2. Exploratory common three-marker Firth penalized Cox regression models.

Endpoint	Variable	HR	95% CI	p Value
OS	DI	4.95	1.26–44.78	0.018
OS	RI	2.33	0.70–11.96	0.182
OS	PI	1.03	0.42–2.70	0.952
LRFS	DI	1.85	0.39–17.83	0.471
LRFS	RI	2.79	0.33–365.43	0.417
LRFS	PI	7.54	0.90–986.29	0.067
DMFS	DI	3.21	1.02–16.00	0.045
DMFS	RI	2.98	0.92–15.10	0.070
DMFS	PI	0.72	0.31–1.73	0.453
PFS	DI	4.12	1.35–20.33	0.010
PFS	RI	3.53	1.12–17.68	0.029
PFS	PI	1.00	0.46–2.31	0.998

Footnote. DI, RI, and PI were entered simultaneously for each endpoint. These models were intended to compare the markers under a common analytical framework and were exploratory. LRFS estimates were highly imprecise because of the small number of local recurrence events. CI, confidence interval; DI, diffuse infiltration; DMFS, distant metastasis-free survival; HR, hazard ratio; LRFS, local recurrence-free survival; OS, overall survival; PFS, progression-free survival; RI, radiological infiltration; PI, pathological infiltration.

Table S3. Exploratory common three-marker models additionally adjusted for pathological surgical margin status.

Endpoint	Variable	HR	95% CI	p Value
OS	DI	4.87	1.23–44.20	0.021
OS	RI	2.43	0.71–12.68	0.170
OS	PI	1.01	0.41–2.68	0.991
OS	R1 margin	1.93	0.20–8.69	0.497
LRFS	DI	1.66	0.33–16.23	0.566
LRFS	RI	4.74	0.44–665.05	0.239
LRFS	PI	6.59	0.77–862.51	0.096
LRFS	R1 margin	5.14	0.48–28.08	0.148
DMFS	DI	2.92	0.92–14.62	0.072
DMFS	RI	3.97	1.16–20.89	0.026
DMFS	PI	0.61	0.26–1.51	0.277
DMFS	R1 margin	6.60	1.18–25.62	0.034
PFS	DI	3.83	1.24–18.96	0.017
PFS	RI	4.86	1.41–26.01	0.009
PFS	PI	0.88	0.40–2.07	0.767
PFS	R1 margin	5.56	1.00–21.31	0.050

Footnote. DI, RI, PI, and pathological surgical margin status were entered simultaneously for each endpoint. These models were exploratory. Only three patients had R1 margins, and LRFS included seven events; corresponding estimates should therefore be interpreted cautiously. CI, confidence interval; DI, diffuse infiltration; DMFS, distant metastasis-free survival; HR, hazard ratio; LRFS, local recurrence-free survival; OS, overall survival; PFS, progression-free survival; RI, radiological infiltration; PI, pathological infiltration.

Table S4. Sensitivity analysis restricted to R0-resected patients.

Endpoint	Model / term	HR	95% CI	p Value
OS	DI adjusted for age, grade, and tumor size	3.73	0.94–33.89	0.064
DMFS	DI adjusted for age, grade, and tumor size	2.90	0.89–14.72	0.079
PFS	DI adjusted for age, grade, tumor size, and RI	3.44	1.09–17.18	0.033
PFS	RI adjusted for age, grade, tumor size, and DI	2.94	0.95–14.61	0.064
LRFS	PI adjusted for tumor size; exploratory	10.31	1.12–1385.04	0.037

Footnote. CI, confidence interval; HR, hazard ratio; LRFS, local recurrence-free survival; DMFS, distant metastasis-free survival; OS, overall survival; PFS, progression-free survival. Analyses were restricted to patients with R0 resection. The LRFS model is exploratory because only seven local recurrence events occurred.

Figure S1. Overall survival according to diffuse infiltration.

Kaplan–Meier estimates of overall survival according to diffuse infiltration (DI) status. Patients with DI-positive tumors had shorter overall survival than those with DI-negative tumors (log-rank $p = 0.019$). Tick marks indicate censored observations. Numbers at risk are shown below the plot.

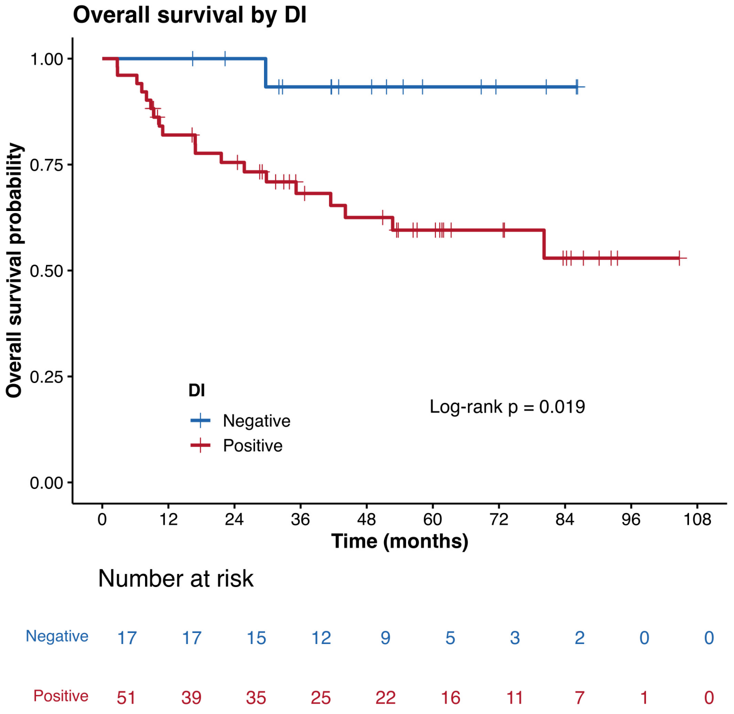

Figure S2. Distant metastasis-free survival according to diffuse infiltration.

Kaplan–Meier estimates of distant metastasis-free survival according to diffuse infiltration (DI) status. Patients with DI-positive tumors had shorter distant metastasis-free survival than those with DI-negative tumors (log-rank p = 0.036). Tick marks indicate censored observations. Numbers at risk are shown below the plot.

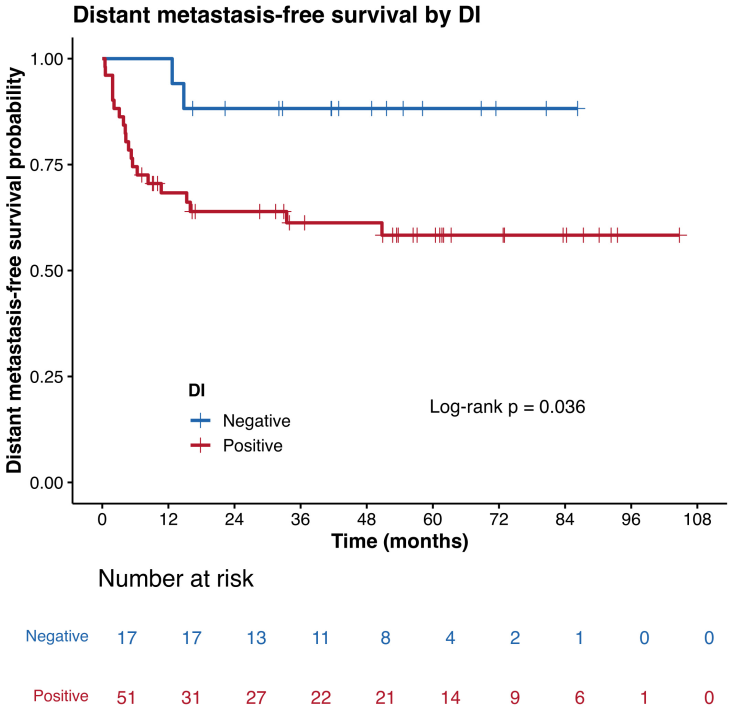

Figure S3. Local recurrence-free survival according to pathological infiltration in patients with R0 resection.

Kaplan–Meier estimates of local recurrence-free survival according to pathological infiltration (PI) status among patients who underwent microscopically margin-negative (R0) resection. Patients with PI-positive tumors had shorter local recurrence-free survival than those with PI-negative tumors (log-rank p = 0.041). Tick marks indicate censored observations. Numbers at risk are shown below the plot.

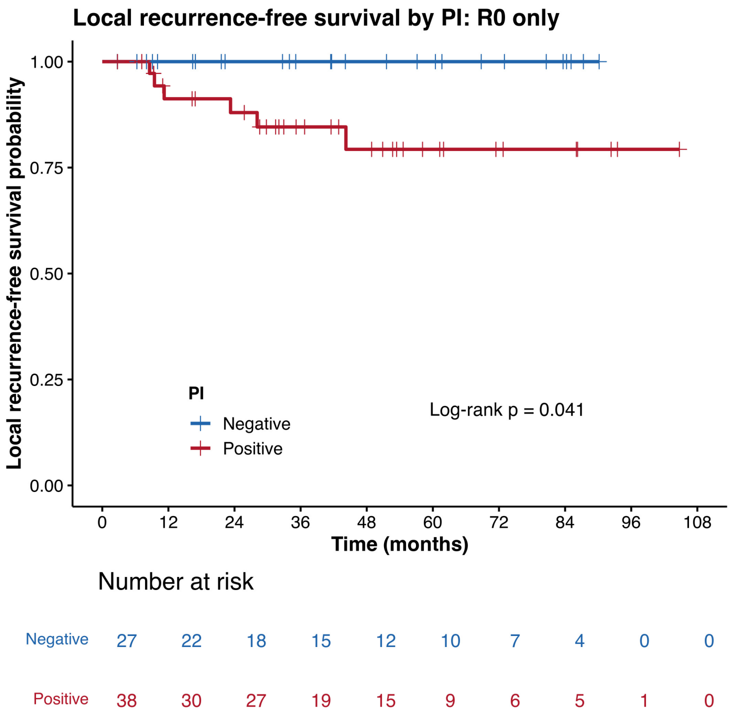

Supplement: Supplementary file 1 [file curroncol-33-00572-s001.zip › curroncol-4546564-supplementary.pdf]
